# Harnessing type I interferon-mediated immunity to target malignant brain tumors

**DOI:** 10.3389/fimmu.2023.1203929

**Published:** 2023-05-25

**Authors:** Juhee Lim, In Kang, Jeongwoo La, Keun Bon Ku, Byeong Hoon Kang, Yumin Kim, Won Hyung Park, Heung Kyu Lee

**Affiliations:** ^1^ Graduate School of Medical Science and Engineering, Korea Advanced Institute of Science and Technology (KAIST), Daejeon, Republic of Korea; ^2^ Department of Biological Sciences, Korea Advanced Institute of Science and Technology (KAIST), Daejeon, Republic of Korea; ^3^ Department of Convergent Research of Emerging Virus Infection, Korea Research Institute of Chemical Technology, Daejeon, Republic of Korea

**Keywords:** type I interferons, brain tumors, glioblastoma, tumor microenvironment, immunotherapy

## Abstract

Type I interferons have long been appreciated as a cytokine family that regulates antiviral immunity. Recently, their role in eliciting antitumor immune responses has gained increasing attention. Within the immunosuppressive tumor microenvironment (TME), interferons stimulate tumor-infiltrating lymphocytes to promote immune clearance and essentially reshape a “cold” TME into an immune-activating “hot” TME. In this review, we focus on gliomas, with an emphasis on malignant glioblastoma, as these brain tumors possess a highly invasive and heterogenous brain TME. We address how type I interferons regulate antitumor immune responses against malignant gliomas and reshape the overall immune landscape of the brain TME. Furthermore, we discuss how these findings can translate into future immunotherapies targeting brain tumors in general.

## Introduction

Brain tumors result in a substantial proportion of cancer-related deaths, with malignant gliomas being the most aggressive and difficult to treat. In adults, glioblastoma (GBM) is the most common primary brain tumor and is highly invasive with very limited treatment options (1). The prognosis for GBM is dismal with a mean survival rate of 14 to 15 months (2). While the current standard of care involves surgical resection, chemotherapy, and radiotherapy, there is a very high probability of recurrence due to intrinsic resistance mechanisms. Thus, GBM and malignant gliomas in general are aggressive and devastating central nervous system (CNS) diseases (1, 3, 4).

Strategies to harness the highly aggressive and heterogenous brain tumor microenvironment (TME) of GBM have been extensively studied. Much research has focused on the role of type I interferons (IFNs) in antiviral immunity since their initial discovery (5). Recently, growing attention has been given to the role of IFNs in antitumor responses against various cancers and their effects in shaping the brain TME. Type I IFNs are a family of cytokines comprised of 13 subtypes of IFNα (IFNα1 - IFNα13), IFNβ, IFNϵ, IFNκ, and IFNω (6). These cytokines can intervene in all phases of cancer immunoediting to eliminate malignant tumor cells and mediate antineoplastic effects against cancerous malignancies (7).

Substantial experimental data point to an indispensable role of type I IFN signaling in tumors. The signal transduction pathway that is initiated upon contact with foreign DNA or RNA plays a key function in regulating anticancer immunosurveillance and regulates intrinsic and extrinsic effects on tumor cells (8, 9). Perturbations in the IFN signaling pathway promote tumorigenesis in sarcomas, melanomas, and brain tumors (10). As a result of type I IFN production, tumor-infiltrating lymphocyte populations that participate in antitumor immunity are activated within the brain TME. There is a growing body of evidence suggesting that CD8^+^ T-cell survival and enhanced cytotoxicity are promoted upon type I IFN production (10). Additionally, tumor-infiltrating dendritic cells (DCs) upregulate the expression of maturation markers and tumor-associated macrophages (TAMs) are polarized into different M1/M2 subtypes according to their respective functions. IFNs not only confer host-protective mechanisms but also promote immunosuppression through their effects on tumor cells. Thus, type I IFNs exert a range of effects on both the immune and non-immune compartments of the brain TME and their contribution to tumor regression (or progression) cannot be ignored.

This review discusses the growing evidence that type I IFNs can either facilitate or impede antitumor immune responses, specifically against brain tumors. The advances that have been made thus far in this field have translated into clinical settings, showing promise for future immunotherapies against malignant gliomas.

## Regulation of canonical type I IFN signaling

Initiation of type I IFN signaling begins with the recognition of pathogen-associated molecular patterns (PAMPs) or damage-associated molecular patterns through pattern-recognition receptors (PRRs) such as toll-like receptors (TLRs) (11). Depending on which TLR is activated, initial stimulation of these receptors triggers an intrinsic downstream signaling pathway that eventually induces the production of type I IFNs. In some cases, oncolytic viruses (OV) can trigger the release of type I IFNs after the identification of PAMPs through PRRs in cancer cells (12).

**Figure 1:**
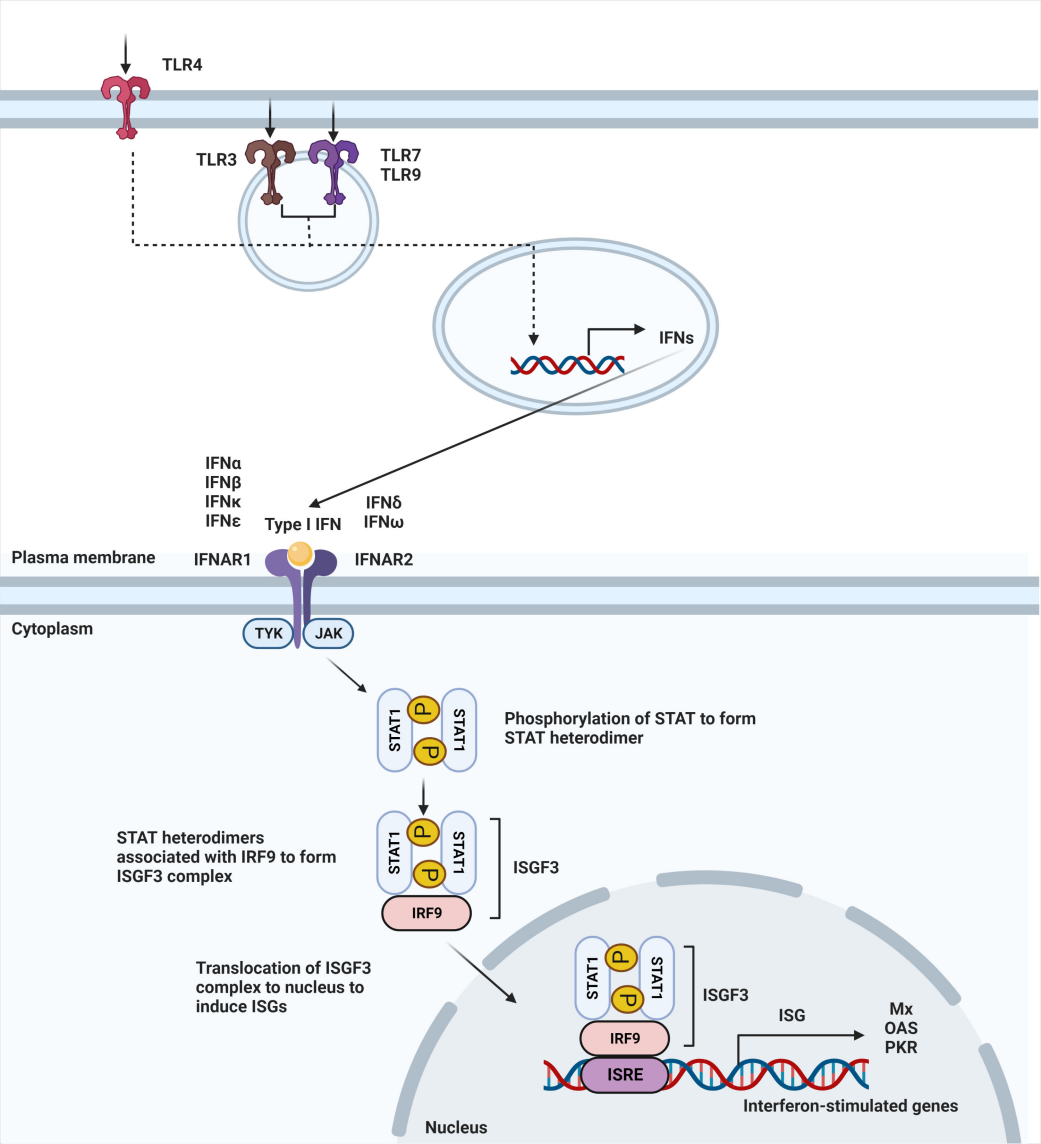
Type I IFN signaling pathway. Secretion of type I interferons starts with stimulation of TLR4 expressed on the cell surface or TLR3, TLR7, and TLR9 that is expressed within endosomes. Stimulation of TLR3, TLR4, TLR7, and TLR9 leads to induction of type I IFN signaling. Upon ligand recognition, triggering of these TLRs activates a downstream pathway that results in the production of type I interferons. Type I interferons, in turn, signal through IFNAR to activate JAK-STAT pathway, leading to dimerization of STAT. Binding with IRF9 results in ISGF3 complex that translocates to the nucleus to bind to interferon-stimulated response elements (ISRE), leading to the induction of interferon-stimulated genes (ISGs).

IFN-α and -β receptor (IFNAR) is ubiquitously expressed on nearly all immune cells (13). The binding of type I IFNs to their heterodimeric receptor consequently triggers activation of the JAK-STAT pathway. IFNAR1 and IFNAR2 are the subunits that make up the transmembrane receptor, with IFNAR2 having a higher binding affinity (14). Upon engagement of this receptor, JAK1 and TYK2 are phosphorylated to activate the dimerization of the STAT1 and STAT2 transcription factors. Dimerization of these STAT proteins leads to the recruitment of IFN regulatory factor 9 (IRF9) to form the ISGF3 complex. Once the ISGF3 complex translocates to the nucleus, it binds to IFN-stimulated response elements (ISREs), DNA sequence motifs that activate the transcription of IFN-stimulated genes (ISGs) (**Figure 1**) (15). These canonically expressed ISGs are referred to as the IFN signature, a hallmark of certain diseases (16).

The absence or downregulation of IFNAR can drive the TME into an immunosuppressive microenvironment that protects malignant tumor cells from cytotoxic T lymphocytes (CTLs), resulting in a poor prognosis in cancer patients (17). The indispensable role of IFNAR is additionally highlighted in a study that showed the downregulation of IFNAR1 promotes melanoma progression (18). Furthermore, the role of IFNAR in antitumor immune responses was highlighted through the use of glioma mouse models that showed reduced infiltration of CTLs and an increase in immunosuppressive Foxp3^+^ regulatory T cells in *Ifnar* ^-/-^ mice with induced gliomas (19).

Interferon signaling has been implicated not only in tumors, but also in aging-associated cognitive decline. Blocking of IFN signaling within the aged brain restored cognitive function and reestablished proper choroid plexus activity, as demonstrated by Baruch et al. (20). Thus, a chronic IFN signature has been shown to be negatively correlated with brain function in the aging brain (20).

## Dual roles of type I interferons in tumors

IFNs are important during the early phases of immune responses when interactions between innate and adaptive immune cells take place (21). Type I IFNs can either restrain or promote tumor growth depending on the duration of the transduced signaling and the associated ISG signature within the TME (22). IFN potentiates immune functions by enabling DCs to cross-prime T cells and restricts tumor development as shown in various solid tumor models. The indispensable involvement of IFNs was demonstrated during early immune rejection of tumors when neutralization of endogenous IFNα/β enhanced the growth of transplanted tumor cells in immunocompetent hosts (23). In addition, type I IFNs can perturb the cancer cycle by obstructing cell cycle stages to prevent tumor cell proliferation. Downregulation of cyclin and cyclin-dependent protein kinase (CDK), proteins that boost the cell cycle, by IFNs consequently inhibit tumor cell proliferation (23). In addition to inhibition of tumor cell proliferation, IFNs can directly induce apoptosis of tumor cells. Mechanisms of IFNα-induced apoptosis include TRAIL-mediated apoptosis, expression of FasL and TNFα, and expression of proapoptotic proteins such as STAT1, STAT6, and TGFβ (24).

**Figure 2:**
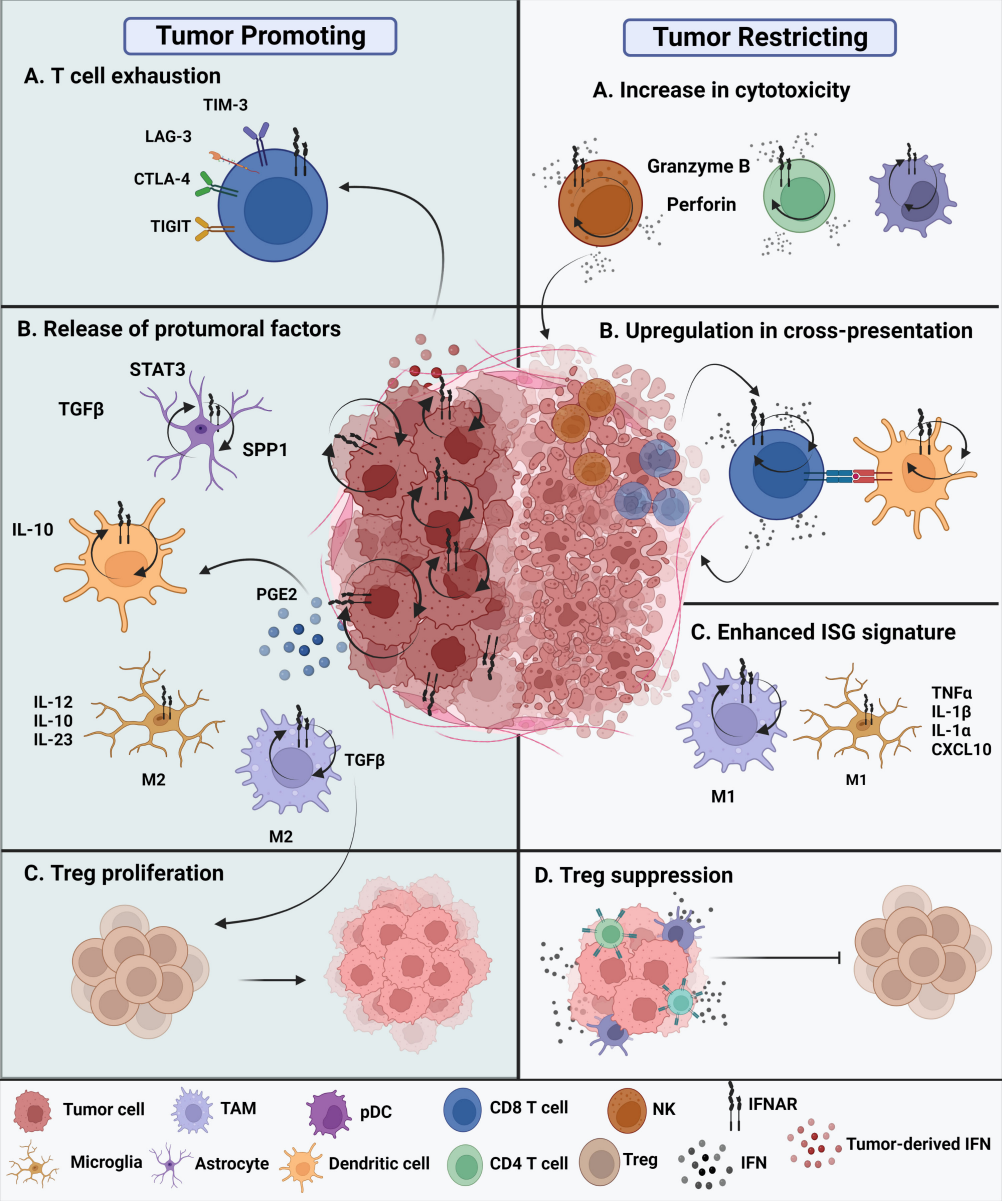
Protumoral and antitumoral roles of type I IFNs in the glioma tumor microenvironment. Tumor promoting roles of IFNs. **(A)** Chronic intrinsic tumor cell IFN signaling leads to dysfunction of T cells, promoting increased expression of exhaustion markers such as TIM-3, LAG-3, CTLA-4, and TIGIT. This ultimately leads to immune checkpoint blockade resistance. **(B)** All cell types within the TME express IFNAR and have type I IFN signaling. The glioma tumor microenvironment promotes the release of protumoral factors. Reactive astrocytes release TGFβ, STAT3, and osteopontin (SPP1) are known to contribute to tumor progression. In addition, release of IL-10 by tumor-infiltrating DCs promote tumor growth along with enhanced TGFβ expression by protumoral M2 TAMs. Tumor-promoting M2 microglia are known to release proinflammatory IL-12, IL-10, and IL-23. **(C)** The nearby release of TGFβ promotes Treg proliferation contributing to a tumor promoting TME. Tumor restricting roles of IFNs. **(A)** Type I IFNs potentiate the cytotoxicity of innate cells, such as NK cells, CD8 T cells, CD4 T cells, and even innate-like pDCs. **(B)** Cross-presentation ability of tumor-infiltrating dendritic cells is enhanced through upregulation of maturation markers, that in turn, enhance CD8 T cell effector responses. **(C)** M1 TAMs and M1 microglia have been suggested to acquire an enhanced ISG signature in the presence of type I interferons in the brain TME. **(D)** The overall secretion of type I IFNs, whether they are tumor cell-derived or host-derived, can either directly or indirectly affect the functions of other nearby immune cells. Thus, these cytokines exhibit a paracrine effect. Secretion of IFNs can suppress Treg function and promote an antitumoral environment.

The current understanding of the role of type I IFNs in malignant gliomas remains confounding (**Figure 2**). On one hand, type I IFNs can inhibit human glioma stem cell growth. IFNβ stimulation of glioma stem cells enriched genes associated with upregulated immune responses and downregulated cell cycle pathways (25). Inhibitory effects of GBM cell growth were also observed following pre-exposure to IFNβ in combination with a cyclin-dependent kinase inhibitor (26). On the other hand, type I IFNs may not always exert protective effects during antitumor immune responses. Experimental evidence suggests that these cytokines can also play deleterious roles in tumors. Prolonged IFN signaling leads to nitric oxide synthase (NOS2) expression, which is associated with increased intratumoral accumulation of regulatory T cells. This, in turn, leads to immune checkpoint therapy resistance. Thus, while IFN signaling is essential for protective functions, constitutive IFN signaling within tumor cells can drive a feedback loop that suppresses antitumor immune responses (8). Additionally, there is evidence that type I IFNs can regulate the dysfunction of T cells. Sumida et al. showed that IFNβ upregulates the expression of PD-1, TIM-3, and LAG-3 exhaustion markers on human T cells. Additional transcriptomic analyses revealed an ISG transcriptional network and identified non-canonical regulators of IFN responses that regulate coinhibitory receptor expression on T cells (27). Additionally, chronic IFN signaling heightens CD8^+^ T cell exhaustion by perturbing lipid metabolism and redox balance, thereby raising oxidative stress (28). The TCF1-Bcl6 axis was shown to repress IFN-mediated T cell exhaustion (29).

To further explore the diverse effects of type I IFNs in malignant brain tumors, there must be an understanding of the overall TME of gliomas and the cellular components that make up the brain tumor immune landscape.

## A look into the tumor microenvironment of malignant gliomas

The glioma TME is highly immunosuppressive and highly complex. The heterogeneity of the GBM TME can be attributed to multiple factors, including genomic driver mutations, differences across tumor subtypes, and the complicated network of tumor and immune cells (30, 31). A fine-tuned look into the immune landscape of the brain TME reveals an intricate network of cellular and molecular interactions. There is a dominance of proliferating tumor cells, a small number of tumor-infiltrating lymphocytes, and a heterogenic population of myeloid cells and microglia that make up much of the TME. Along with the immune compartment, the brain TME is also characterized by blood vessels, astrocytes, and the extracellular matrix (32). Type I IFNs can function as immune-activating components within the heterogenic and hypoxic brain TME or as sabotaging agents that promote protumoral responses against gliomas. Type I IFNs exert their effects on immune cells either directly via IFNAR or indirectly through the induction of chemokines that recruit other immune cells to the tumor site (33).

Another hallmark feature of the brain TME is the very low level of oxygen. Hypoxia enhances the aggressiveness of tumors and is driven by oxygen consumption by tumor cells (34). Due to the hypoxic nature of the brain TME, there is increased tumor cell invasion and reduced cytokine production, proliferation, and exhaustion of effector cells (35). IFN signaling is downregulated in hypoxic microenvironments due to repressed transcription and reduced chromatin accessibility, which occurs in a HIF1/2α-independent manner (36). A still unanswered question is how the hypoxic nature of the brain TME affects the overall IFN signature specifically in the context of malignant gliomas.

## Effects of type I interferons on non-immune cell components in the brain tumor microenvironment

### Interferon regulation of glioma tumor stem cells

While numerous studies have focused on IFNs produced by immune cells in the TME, glioma stem cells also retain intrinsic type I IFN signaling that enables immune evasion from host cells. The Gl261, CT2A, and SMA-560 glioma cell lines uniformly express IFNAR1 and IFNAR2. Thus, autocrine type I IFN signaling is likely intact within these tumor cells lines, and ablation of IFNAR in the Gl261 cell line affected *in vitro* and *in vivo* tumor cell growth (37). Furthermore, glioma stem cells evade host type I IFN suppression through the downregulation of STAT1 mediated by the MBD3/NuRD complex (38). Inactivation of STAT signaling provides a means of escape for glioma tumor cells from IFN suppression. Another study showed that glioma stem cells exhibit a differential cell-intrinsic type I and type II IFN signature. This cell-intrinsic IFN signaling among glioma stem cells regulates tumor cell proliferation and correlates with mesenchymal phenotypes (39). In addition to the effects of tumor IFN signaling, other cell-intrinsic characteristics of tumor cells affect tumor immunogenicity. For example, high levels of PD-L1, expressed by cancer cells, inhibit cytotoxic type I IFN responses and sustain chronic responses that enhance the IFN-related damage resistance signature (IRDS). This, in turn, prevents cancer cell death (40).

Apart from tumor intrinsic IFN signaling, exogenous type I interferons are able to inhibit glioma cells. IFNβ suppressed growth of glioma stem cells by downregulating cell proliferation and ribosome pathways (25). Also, pulse stimulation of GSCs with IFNβ reduced sphere formation capacity and migratory signature (41). In addition, treatment of IFNβ sensitized GSCs to temozolomide, which was associated with upregulation of XAF1 and TRAIL death ligands (42). Altogether, tumor-derived interferon signaling can promote immune evasion from host cells while exogenously administered interferons can inhibit GSC growth.

### Astrocytes: the most abundant cell type in the central nervous system

Astrocytes account for approximately half of the cells in the brain and contribute to proper CNS development. Astrocytes maintain the overall structure of the blood-brain barrier through interactions with pericytes and endothelial cells (43, 44). Within the brain TME, astrocytes interact with tumor cells via gap junctions, ion channels, and the release of gliotransmitters and extracellular vesicles (45). During GBM development, astrocytes are activated and the TME polarizes reactive astrocytes to tumor-supporting glial cells. Reactive astrocytes in the GBM compartment produce tumor-promoting factors such as TGFβ and STAT3 that promote tumor metastasis (45). A comparison of high- and low-grade gliomas revealed a subpopulation of astrocytes expressing high levels of osteopontin (SPP1) in high-grade gliomas, suggesting a correlation with poor survival among glioma patients (46, 47).

Type I IFN signaling in astrocytes regulate immune responses in the CNS through cell-intrinsic and extrinsic manners. For example, in viral neuroinfections, astrocytes produce type I IFNs (48), indicating their role as IFN producers in the CNS and potentially in the brain TME as well. Type I IFN signaling is critical in astrocytes for synaptic plasticity and memory formation mediated through the astrocytic glutamate-aspartate transporter (GLAST). IFNAR loss in astrocytes impairs proper hippocampus and cognitive functions through glutamate uptake modulation (49). Tumor-associated astrocytes in the GBM TME are anti-inflammatory and are enriched in gene sets associated with JAK/STAT pathway activation and IFNα and IFNγ responses. These tumor-associated astrocytes were later suggested to mediate specific re-programming of microglia cells (50).

### Resident microglia

Resident microglia are brain tissue-resident macrophages that make up the main immune cell populations in the brain. These cells participate in various physiological processes in the brain including neurogenesis and axon growth (51). Recent advances in imaging mass cytometry have provided insight into the spatial organization of the GBM TME, revealing that tissue-resident microglia and monocyte-derived macrophages comprise the dominant immune populations across human GBM samples (52). There is a heterogenous population of these glioma-associated microglia (GAMs) that are divided into antitumoral M1 and immunosuppressive M2 phenotypes (53). M1 GAMs are distinguished by their ability to secrete proinflammatory cytokines such as IL-23, IL-12, IL-6, and IL-1β along with the production of reactive oxygen species (ROS). Expression of STAT1 by M1 GAMs promotes their antitumoral function due to the tumor suppressor activities associated with STAT1 expression (54). M2 GAMs, on the other hand, are characterized by low expression levels of MHC II, IL-12, and IL-23 and secretion of anti-inflammatory cytokines such as TGFβ and IL-10, which are important for the prevention of destructive immune responses (51).

Type I IFNs allow microglia to exert multifaceted roles in the CNS, including maintaining homeostasis and recovering from injuries (55). The overall immune landscape during the early stages of GBM is predominated by M1 proinflammatory GAMs, characterized by an upregulation of genes associated with proinflammatory processes such as *Tnf*, *Il1b*, *Il1a*, and *Cxcl10* (56). Despite the growing evidence of GAM functions in the brain TME, there are still unanswered questions regarding how type I IFNs affect microglial function in CNS diseases and, in particular, gliomas. Attempts to narrow this knowledge gap can be seen in a recent study demonstrating that peripherally derived IFNα can directly transduce signaling effects across the blood-brain barrier on resident microglia. Direct microglia IFN signaling is involved in the transcriptomic changes that are seen in the brain parenchyma as a result of peripherally administered IFNα (57). In addition, adenoviral-mediated IFNβ expression in an orthotopic Gl261 GBM model showed an activated microglia phenotype, in correlation with increased tumor cell death and improved mouse survival (58).

## Effects of type I interferons on immune cell components in the brain tumor microenvironment

### Inflammatory monocytes

Growing evidence suggests that monocytes play a role in the development and progression of cancers, including GBM. The current understanding regarding the effects of type I IFNs on monocytes in cancerous settings is based on its immunostimulatory effects. The frequency of suppressive monocytes is often associated with resistance to immune checkpoint blockade (ICB) therapy. Cancer cell-derived type I IFNs polarize a subset of intratumoral monocytes to function as immunostimulatory mediators. Type I IFN signaling is necessary for tumor-associated monocytes to drive CD8^+^ T-cell proliferation as well as restrain the immunosuppressive activities of different monocyte subsets (59). In addition, a recent study conducted by Zemek et al. showed that inflammatory monocytes are the primary source of IFNβ in responding tumors and that type I IFN signaling within tumor-infiltrating monocytes contributes to T cell expansion (60). In addition, Ochoka et al. suggested that monocyte/macrophage clusters show a higher expression of type I IFN-related genes in relation to other cell clusters, indicating that monocytes are affected by type I IFN signaling within the GBM TME (61)

In malignant gliomas, circulating monocytes migrate to the tumor niche and differentiate into either TAMs or tumor-associated microglia (62). A reduction in classical and non-classical monocytes was observed in glioma patients compared to healthy volunteers. Non-classical monocytes from the glioma patients exhibited a proinflammatory cytokine profile (TNFα and IL-12), suggesting a role of monocytes in antitumor immune responses against gliomas (63). Monocytes have become potential therapeutic candidates as IFN gene-delivery vehicles into the brain TME. Tie-2 expressing monocytes, due to their homing ability to the TME, are efficient cellular vehicles for the delivery of IFNs via lentiviral transduction methods (64, 65).

### Tumor-associated macrophages

TAMs make up a prevalent portion of the heterogeneous population of immune cells in the brain TME. Depending on their functions, TAMs are divided into M1 and M2 subtypes. In general, M1 TAMs carry out antitumoral functions that facilitate Th1 responses and secrete proinflammatory cytokines such as TNFα. M2 TAMs promote tumor progression through the secretion of anti-inflammatory cytokines, such as TGFβ, that promote Treg and tumor cell proliferation (66). These M1 and M2 TAMs can also be distinguished based on whether they reside in the tumor periphery or the hypoxic tumor core, respectively (67).

A recent study identified a subset of proinflammatory CD169^+^-expressing TAMs that contribute to antitumor immune responses against GBM. These cells are enriched in gene sets for responses to IFNα, suggesting that CD169^+^ TAMs are activated by type I IFNs (68). Transcriptomic profiling of myeloid cells in Gl261 glioma-bearing mice shows that glioma-associated macrophages are enriched in gene sets related to responses to IFNβ and several upregulated gene sets that overlap with activated microglia populations (61). Taken together, heterogenous populations of TAMs in high-grade glioma TMEs exhibit a type I IFN signature that plays a role in shifting TAMs toward protumoral or antitumoral myeloid cells.

### Tumor-infiltrating DCs

Several studies have detailed the roles of DCs within the brain TME. Experimental data suggest an interplay between DCs, microglia, macrophages, and T cells (69). The main role of tumor-infiltrating DCs is to take up and present tumor-associated antigens to CTLs (70). In a microenvironment where there is a high level of immunosuppression, such as within the brain TME, DCs are usually in an inhibitory or immature state (71). Differentiation of DCs to other subsets is inhibited by immunosuppressive cytokines that are secreted by glioma cells. For example, prostaglandin E2 is produced by glioma cells that, in turn, promotes IL-10 production by DCs to inhibit effector T cell responses (71). In another study, the role of the cDC1 subset was characterized during an endogenous immune response to brain tumors. cDC1s are required for neoantigen-specific responses and responses to ICBs in syngeneic models of GBM (72).

One of the main immunostimulatory actions of type I IFNs on DCs is to enhance their cross-presentation ability to CD8^+^ T cells. Transcriptional profiling of intratumoral DCs revealed that tumor-infiltrating DCs exist in different functional states. Activation of CD11b^+^ DCs is characterized by an ISG signature, and the “ISG-DCs” can activate CD8^+^ T cells. Sustained IFN signaling derived from local tumor cells promotes this ISG-like state within CD11b^+^ intratumoral DCs. Thus, these DCs present tumor-derived peptides via MHC I dressing (73).

### Plasmacytoid dendritic cells

The antitumoral activities of plasmacytoid DCs (pDCs) have been studied in various solid tumor models. The hallmark of pDCs is their ability to produce large amounts of type I IFN. Their role in antiglioma immunosurveillance was described in a study that used transgenic mice to deplete pDCs. Using a syngeneic murine model of glioma, pDC depletion leads to increased survival of mice bearing intracranial Gl261 tumors. Additionally, there is a notable change in immune cell infiltrates. Analysis of the immune cell profile revealed a decrease in immunosuppressive regulatory T cells upon pDC depletion in Gl261 tumor-bearing mice (74), suggesting a role of pDCs in antitumor immunity against GBM.

### Cytotoxic T lymphocytes

CTLs are the main executors in the regulation of antitumor immune responses and make up the backbone of cancer immunotherapy (75). These effector T cells have been extensively studied in viral infections, and their detailed roles in antitumor responses against brain tumors have recently gained much attention. A positive GBM prognosis is associated with a high ratio of CD8^+^ T cells to CD4^+^ T cells. Among CD8^+^ tumor-infiltrating lymphocytes, a subset of CD8^+^Tbet^+^ cells indicates the presence of activated proliferating CD8^+^ T cells within the TME (76). Although cytotoxic CD8^+^ T cells are known for their effector functions, these cells may exist in a dysfunctional state when exposed to the brain TME. Chronic immune activation leads to terminal CD8^+^ T-cell exhaustion mediated by IL-10 release from the myeloid compartment of the TME (77). Other factors that render T cells dysfunctional in the brain TME include high levels of hypoxia (78), limited glucose availability (79), and production of oncometabolites (80). Thus, the role of CD8^+^ effector T cells cannot be ignored during antitumor immunity against brain tumors. Various cytokines and chemokines mediate and regulate the activity of these CTLs, including type I IFNs.

The immunomodulatory effects of type I IFNs on CD8^+^ T cells are pleiotropic (81). Type I IFNs directly enhance the cytotoxic functions of CD8^+^ T cells, stimulating the production of IFNγ and TNFα and enhancing the secretion of granzyme A, perforin, and granzyme B. IFNs with IL-2 can function as a third signal for naïve CD8^+^ T-cell differentiation (82) and protect T cells from natural killer (NK) cell-mediated attack (83). T cells that lack IFNAR are more susceptible to NK cell-mediated attack through the expression of natural cytotoxicity triggering receptor 1 (NCR1) (84). Ablation of IFNAR in mouse models further supports the role of type I IFNs in the pathogenesis of gliomas. Glioma-induced *Ifnar1* ^-/-^ mice show decreased infiltration of CD8^+^ T cells and reduced potency in their cytotoxic functions (19).

### Emerging cytotoxic CD4^+^ T cells

CD4^+^ T cells are differential coordinators of adaptive antitumoral immunity and are best known for their helper functions (85). Once stimulated by antigens presented by MHC II, different helper T cell subsets orchestrate the direction of the immune response through cytokine secretion (86). Helper CD4^+^ T cells typically promote CTL function through the activation of DCs and regulate the myeloid compartment (85). The role of CD4^+^ T cells in regulating antitumor immune responses has been overlooked due to the clinical success of CD8^+^ Tcell-based immunotherapies, and only recently has the importance of CD4^+^ T cells in driving antitumor immunity been recognized. CD4^+^ T cells have been implicated in ICBs in brain tumors. CD4^+^ T-cell deficiency drives CD8^+^ Tcell exhaustion leading to unresponsiveness to PD-1 blockade, as demonstrated through CD4 depletion in a Gl261-induced glioma mouse model (87, 88).

Interestingly, there has been recent evidence that similar to cytotoxic CD8^+^ T cells, cytotoxic CD4^+^ T cells retain antitumoral activity (89). CD4^+^ CTLs, similar to their CD8^+^ CTL counterparts, express granzymes and perforins to carry out tumor-killing functions (89). The direct cytotoxic potential of CD4^+^ T cells in the TME has been shown in human cancers where tumor-specific CD4^+^ T cells portray similarities to classical CTLs. Transcripts for cytolytic-related molecules, such as IFNγ, CCL4, and CCL5, are enriched in these cytotoxic CD4^+^ T cells (90). Thus, CD4^+^ CTLs may play an important role in antitumor immunity against gliomas.

CD4^+^ CAR T cells targeting GBM-associated IL-13 receptor α2 outperform their CD8^+^ CAR T cells counterparts in antitumor activity and exhibit less activation-induced exhaustion features (91). Increased expression of granzyme A, granzyme B, and perforin by intratumoral CD4^+^ CTLs in gliomas suggests these cells to be a positive prognostic marker for glioma survival (89). Conversely, CD4^+^ T cells can also be negatively associated with ICB therapy. Transcriptomic analysis of tumor-specific CD4^+^ T cells revealed a type I IFN response signature with an upregulated exhaustion signature characterized by increased PD-1 levels (92).

### Regulatory T cells

Regulatory T cells (Tregs), a subset of CD4^+^ T cells, are usually characterized by FOXP3^+^ expression. CD4^+^CD25^high^FOXP3^+^ Tregs function in maintaining self-tolerance, and previous studies have demonstrated their contribution to the suppression of antitumor immune responses (93). Tregs, in addition to M2-like macrophages/microglia, predominate the suppressive immune cell populations within the GBM TME, contributing to ICB resistance and dampening CTL responses (94). Evidence strongly suggests that increased Treg populations are associated with higher histological grades in gliomas, with a significant amount of regulatory T cell infiltration in GBM (95). Immunosuppressive functions of Tregs are perturbed upon αPD1 treatment as was shown using a GBM model. Tregs exhibit an anergic phenotype characterized by the expression of CD73 and FR4. These regulatory T cells express glucocorticoid-induced tumor necrosis factor (GITR) that, when blocked, enhances CD4^+^ effector functions and reduces the immunosuppressive effects of Tregs. The combination of αGITR and αPD1 essentially converts GBM Tregs to Th1-like effector cells (94). In addition, the depletion of Tregs in the TME is associated with improved survival. Administration of the IL-2Rα antibody daclizumab to GBM patients depletes Tregs leading to enhanced antitumor responses (96).

Thus, methods to suppress Treg function within the brain TME may result in a better prognosis for GBM survival, and type I IFNs may provide leverage to overcome Treg-mediated immunosuppression. Rather than a direct inhibitory effect, type I IFNs may indirectly render Tregs dysfunctional through chemokine regulation. Intratumoral IFNα gene delivery into tumors enhances antitumor immune responses by reducing infiltration of regulatory T cells and production of IL-6, a Treg-inhibiting cytokine (97). Another indirect effect of type I IFN-mediated Treg suppression occurs when intratumoral IFNα delivery downregulates tumor expression of CCL17 that trafficks Tregs to tumor centers (93).

### Natural killer cells

NK cells are the innate counterpart of CTLs due to their ability to lyse malignant cells (98). NK cells can kill cancerous cells independently of T cells through secretion of granules containing membrane-disrupting proteins and granzymes. In a recent study, NK cells represented the highest percentage of total infiltrating immune cells in brain cancer, suggesting an indispensable role for these cells (99). NK cells can alter the phenotype of glioma stem-like cells and reduce chemotherapy resistance (100), which is important when considering future immunotherapy options for brain tumors. Despite the immune-activating effects of NK cells, they can also be rendered dysfunctional in the GBM TME (101). For instance, tumor growth factors (TGFβ) from the sera of glioma patients downregulates the expression of NKG2D on NK cells and CD8^+^ T cells, rendering them less efficient in killing tumor cells (102). Thus, attempts to restore NK cell functions are being explored in emerging NK cell-based therapies. Single-cell RNA sequencing analysis of infiltrating NK cells in a Gl261 syngeneic glioma model revealed the downregulation of IFN genes and reduced expression of activation markers (103), suggesting a role for type I IFNs in NK cells in brain tumors.

NK cells are directly activated by type I IFNs resulting in enhanced cytotoxic functions, production of inflammatory cytokines, or crosstalk with antigen-presenting cells (84, 100, 104). NK cell-mediated tumor surveillance is hindered when IFNAR is abolished among these cells, hindering their cytotoxic capabilities as demonstrated in lymphoma and melanoma models (105). There are still unsolved questions concerning the detailed mechanisms of how IFNs regulate NK cells within the brain TME.

## Type I interferons in brain tumor immunotherapy

### Recombinant interferon therapy

**Figure 3:**
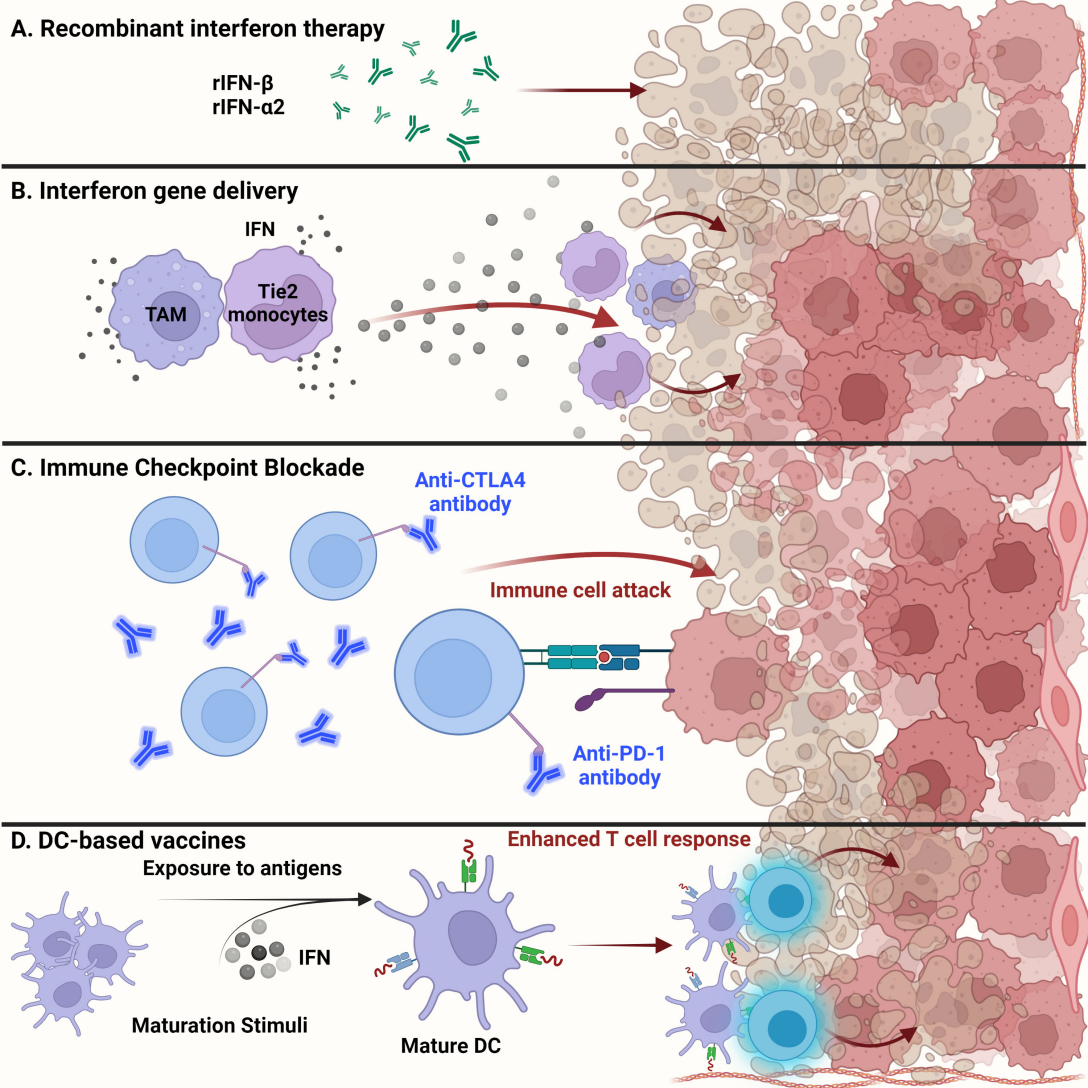
Glioma targeted therapy. **(A)** Apart from current standard of care, administration of recombinant IFNβ and IFNα2 has been approved by the FDA for glioma treatment. Usually used as adjuvants, antitumor activity of these recombinant proteins has been shown in several clinical studies and examined for overall systemic toxicity. **(B)** Engineering of myeloid cells to deliver interferons to the glioma tumor microenvironment has shown therapeutic potential. Recent techniques have leveraged the ability of tumor-associated macrophages and Tie2-expressing monocytes to reach the TME and release type I interferons, inducing glioma cell dysfunction. **(C)** Administration of anti-PD1 monoclonal antibodies to prevent T cell deactivation has shown little success in gliomas than expected. This could be due to the cell intrinsic type I IFN signaling within tumor cells that contribute to ICB resistance. **(D)** Dendritic cell-based vaccines alleviate the immunosuppression of the glioma TME by enhancing T cell responses. Exposure to tumor antigens and pulsing of interferons to *ex vivo* isolated immature DCs leads to their maturation and upregulation to potentiate effector T cell responses against malignant glioma tumor cells.

Exogenous administration of recombinant IFNs is used for treating a range of cancers including hairy cell leukemia, B- and T-cell lymphomas, and solid tumors, such as melanoma and renal cell carcinoma (**Figure 3A**). Dating as far back as 1986, recombinant IFNα received United States Food and Drug Administration (US FDA) approval for treating hematological malignancies, becoming the first therapeutic drug to treat cancer patients (97, 106, 107). Since then, many clinical trials have shown the efficacy of using recombinant type I IFNs in treating patients with solid tumors (106). The efficacy of recombinant IFN therapy has been demonstrated in a randomized clinical trial involving high-grade glioma patients. The overall survival was improved upon treatment with temozolomide and IFNα, demonstrating the benefits of combination treatment over the conventional use of temozolomide alone (108). In addition, the combination of IFNα with BCNU and radiation therapy showed increased overall survival among high-grade glioma patients, thus demonstrating the safe and feasible use of IFNs with other cancer therapies (109).

Recombinant IFN therapy is not a new concept, yet this type of immunotherapy has its setbacks. Virtually all patients treated with recombinant IFNα experience adverse side effects during the course of treatment. Administration of recombinant IFNs can provoke several reactions including cardiovascular, respiratory, endocrine, and metabolic problems (110). Thus, although there has been some success in treating solid cancers including gliomas, the adverse reactions associated with this type of treatment call for a safer and more efficient method for use of IFNs in immunotherapies. Lessons from clinical trials have shown that there are still unresolved issues that need to be addressed. These include the optimal dose-scheduling of IFN treatment. It is important to understand what levels are required to reach optimal interaction with radiation therapy to optimize treatment (111). In addition, optimizing the scheduling of IFN treatment in regards to duration and timing is needed in order to avoid prolonged damage associated with radiosensitization (111).

Though not necessarily explored in gliomas, alternative strategies to overcome the limitations of recombinant interferon therapy have been explored by fusing tumor antigens with interferons in different tumor models (112, 113). Research into improving recombinant interferon therapy in gliomas has not yet been extensively studied. But given the success of combining tumor targeting antibodies with modified forms of interferons in other solid tumors, similar techniques may also be applied to treat gliomas.

### Targeted delivery of interferons to tumor microenvironments

Type I IFNs have broad implications in various physiological processes, especially in the context of immune cells. Thus, given the powerful functions of these cytokines, there have been many recent attempts to harness them as therapeutic drugs. Yet the pleiotropic effects and systemic toxicity that occur when using IFNs as therapeutic drugs have been major hurdles (114). Thus, novel approaches for delivering IFNs to the TME have been explored.

Recent advances in gene therapy show that targeted delivery of immunoactivating cytokines into the TME can endure and overcome TME immunosuppression, while tightly regulating the expression of the delivered cytokines (**Figure 3B**). Birocchi et al. demonstrated that the inducible release of IFNα into the GBM TME perturbed tumor growth and essentially reprogrammed the immune microenvironment toward a proinflammatory and antitumoral state. This reprogrammed immune landscape was characterized by proinflammatory TAMs that exhibited a gene signature associated with poor prognosis in human GBM. This inducible system was able to limit overall systemic toxicity by tightly regulating the release of IFNα into the TME (65). Additionally, Palma et al. showed that angiopoietin receptor Tie2-expressing monocytes have a unique capacity to home to tumors. Therefore, using a lentiviral vector system, these monocytes were engineered to express IFNα and were delivered to the GBM TME, where improved antitumor activity against brain tumors was observed (64).

Other novel approaches to deliver type I IFNs into tumors include arming PD-L1 antibodies with IFNα to overcome PD-L1-mediated immune suppression, while at the same time, increasing antigen cross-presentation to enhance T cell activation (115). Although this technique is therapeutically beneficial in advanced tumors, such as colon cancer and lymphomas, this approach has not been explored in brain tumors.

### Immune checkpoint blockade therapy

In general, recent breakthroughs in cancer therapy using ICB have attracted significant attention. Recent success in ICB therapy targeting PD-1 and CTLA-4 has provided a breakthrough in the field of immunotherapy (**Figure 3C**) (116). Despite some clinical success, however, ICB has not been fully effective in treating high-grade gliomas such as GBM. The immune checkpoint receptors PD-1 and CTLA-4 are expressed on the surfaces of T cells and inactivate T cells once in contact with tumor cells (116). Engagement of PD-1 with its ligands PD-L1 and PD-L2 maintains peripheral tolerance and compromises antitumor immunity (117). Blocking the interaction between these immune checkpoint receptors and tumor cells, thereby preventing T cell inactivation, is the essence of ICB therapy. However, three controlled trials using the monoclonal anti-PD1 antibody nivolumab have failed to improve survival among GBM patients (118). Resistance to ICB therapy, in part, can be credited to sustained intrinsic IFN signaling among tumor cells. Mechanisms of ICB resistance can also be attributed to PD-L1 independent adaptive resistance that is associated with type I IFN gene expression. As demonstrated by Benci et al., PD-L1 independent resistance is essentially orchestrated by chronic IFN signaling (119). Therefore, new potential biomarkers to evaluate the responsiveness of ICB among GBM patients are needed. The biomarkers of ICB resistance may include PD-L1 positivity on tumor and immune cells, an IFNγ gene signature, and the level of IFN signaling that exists within cancerous cells (118). The combined use of ICB and exogenous IFN administration may perhaps improve the clinical efficacy against brain tumors.

### Dendritic cell-based vaccines using type I interferons

DCs have gained interest in their use as therapeutic cancer vaccines. Due to the ability of DCs to process and present antigens to CTLs and helper T cells, DC vaccines exploit this function for therapeutic use (**Figure 3D**). In general, patients are vaccinated with tumor-associated antigen (TAA)-loaded DCs to initiate an antitumoral T cell response (120). These DCs have either been generated from monocyte precursors or CD34^+^ hematopoietic precursors and expanded *in vivo* (121). Increased cytotoxic T cell infiltration with reduced levels of TGFβ in brain tumor regions were seen in dendritic cell-vaccinated patients (122). Combination of autologous tumor lysate-loaded dendritic cell therapy combined with standard therapy showed an improvement in survival of patients with recurrent glioblastoma (123).

In addition, several studies have demonstrated that type I IFNs markedly enhance the maturation, proliferation, and activation of DCs. These so-called ‘IFN-DCs’ have been implicated in high-grade gliomas. LPS-stimulated IFN-DCs from high-grade glioma patients exhibit high levels of the costimulatory molecule CD86 and MHC II antigens, similar to donor IFN-DCs (124). Thus, IFN-DCs can be promising therapeutic candidates to use in DC-based vaccines to treat GBM.

## Future perspectives of type I IFN therapy

Although some treatment options have been met with clinical success, preclinical and clinical studies that have been conducted thus far have helped raise awareness to important questions that need to be addressed to optimize type I IFN therapy. For instance, it may be important to determine which interferon subtype is most effective when used with radiation therapy (111). IFNα and IFNβ have mostly been used thus far in demonstrating the efficacy of IFN therapy. A comparison of various interferon subtypes may be helpful in determining the efficacy of IFN therapy with radiation therapy (111). Thus, according to Goedegebuure et. al, establishing the overall IFN status prior to treatment by analyzing tumor cell IFN receptor expression may add more value to IFN therapy (111). In addition, patient status before IFN treatment may likely differ, depending on the person’s overall health status and medical history. All of these factors may likely influence their response to IFN treatment, and exploring ways to optimize treatment for each patient is a hurdle to be overcome.

## Closing remarks

Type I IFNs have long been recognized as cytokines that exert a broad range of effects. It is becoming increasingly clear that IFNs can function as either potent stimulators of antitumor immunity or enhancers of tumor progression. Because the brain TME is highly immunosuppressive and heterogenic, dissecting the cellular components of the TME is critical for understanding the immunologic interactions that occur. Among these interactions, type I IFNs can stimulate tumor-infiltrating lymphocytes to promote antitumor immune responses or to induce resistance to immunotherapy. While much research remains to be done regarding the detailed roles of type I IFNs in brain tumors, advances in treatment options centered around targeted IFN delivery and ICB point to a hopeful future for treating malignant gliomas.

## Author contributions

JHL, IK, JWL, KBK, BHK, YK, WHP, and HKL wrote the manuscript. All authors contributed to the article and approved the submitted version. 
